# Enhanced Cycling Performance of Rechargeable Zinc–Air Flow Batteries Using Potassium Persulfate as Electrolyte Additive

**DOI:** 10.3390/ijms21197303

**Published:** 2020-10-02

**Authors:** Ramin Khezri, Soraya Hosseini, Abhishek Lahiri, Shiva Rezaei Motlagh, Mai Thanh Nguyen, Tetsu Yonezawa, Soorathep Kheawhom

**Affiliations:** 1 Department of Chemical Engineering, Faculty of Engineering, Chulalongkorn University, Bangkok 10330, Thailand; ramin.k@chula.ac.th (R.K.); soraya.h@chula.ac.th (S.H.); 2 Department of Chemical Engineering, Brunel University London, London UB8 3PH, UK; abhishek.lahiri@brunel.ac.uk; 3 Department of Chemical Engineering, Faculty of Engineering, Universiti Putra Malaysia, Selangor 43300, Malaysia; shiva.rezaei@student.upm.edu.my; 4 Division of Materials Science and Engineering, Faculty of Engineering, Hokkaido University, Hokkaido 060-8628, Japan; mai_nt@eng.hokudai.ac.jp (M.T.N.); tetsu@eng.hokudai.ac.jp (T.Y.); 5 Institute for the Promotion of Business-Regional Collaboration, Hokkaido University, Sapporo 001-0021, Japan; 6 Research Unit of Advanced Materials for Energy Storage, Chulalongkorn University, Bangkok 10330, Thailand

**Keywords:** zinc–air batteries, sulfur-containing additive, electrolyte additive, cycling performances, electrochemical characterization

## Abstract

Zinc–air batteries (ZABs) offer high specific energy and low-cost production. However, rechargeable ZABs suffer from a limited cycle life. This paper reports that potassium persulfate (KPS) additive in an alkaline electrolyte can effectively enhance the performance and electrochemical characteristics of rechargeable zinc–air flow batteries (ZAFBs). Introducing redox additives into electrolytes is an effective approach to promote battery performance. With the addition of 450 ppm KPS, remarkable improvement in anodic currents corresponding to zinc (Zn) dissolution and limited passivation of the Zn surface is observed, thus indicating its strong effect on the redox reaction of Zn. Besides, the addition of 450 ppm KPS reduces the corrosion rate of Zn, enhances surface reactions and decreases the solution resistance. However, excess KPS (900 and 1350 ppm) has a negative effect on rechargeable ZAFBs, which leads to a shorter cycle life and poor cyclability. The rechargeable ZAFB, using 450 ppm KPS, exhibits a highly stable charge/discharge voltage for 800 cycles. Overall, KPS demonstrates great promise for the enhancement of the charge/discharge performance of rechargeable ZABs.

## 1. Introduction

Zinc–air batteries (ZABs) are promising energy storage systems (ESSs), being cost-effective and eco-friendly and having high energy densities [1,2,3,4]. However, rechargeable ZABs, due to poor cycling performance, have not been widely used on a commercial scale [5,6,7]. Numerous issues have been raised as possible reasons for the low electrical rechargeability of ZABs, including Zn corrosion, Zn oxide (ZnO) precipitation on the Zn anode and dendritic growth of Zn [8,9]. Such issues are responsible for a decrease in efficiency and poor cycling performance. These drawbacks can be mitigated by improving the air cathode configuration [10,11,12], modifying the Zn anode structure [13,14,15] or using electrolyte additives [16,17,18].

In the electrochemistry of batteries, electrolytes play an important role. Electrolytes help to transport hydroxide ions during the operation of ZABs. The composition of electrolytes significantly influences the capacity and stability of ZABs [19]. Electrolytes also determine the energy and power density of the batteries. Alkaline electrolytes such as concentrated potassium hydroxide (KOH) are generally used in the operation of ZABs, owing to their high ionic conductivity, fast electrochemical kinetics and good solubility of Zn [20,21,22]. 

The use of KOH in ZABs raises several problems, viz. the growth of dendrite during charging and the corrosion of the Zn anode due to the hydrogen evolution reaction (HER) [23]. In addition, one of the most challenging issues of using KOH in ZABs is the accumulation and precipitation of discharged products on the active Zn surface, leading to an undesirable passivation effect and the degradation of battery performance [16]. Therefore, the selection of a suitable electrolyte is crucial in improving cycling performance and battery efficiency. Recently, attempts have been made to modify alkaline electrolytes to enhance the performance of ZABs. Many approaches have been proposed, focusing on the use of inorganic additives, organic solvents (such as surfactants) and polymer gels [5,17,24,25].

Sulfur-containing compounds, namely ethylene sulfide, sodium dodecyl sulfate (SDS) and dimethyl sulfoxide (DMSO), as additives to electrolytes, have been investigated by a few researchers [16,18,26]. It is noted that sulfur compounds are relatively easier to reduce than other components in electrolytes and can enhance redox activity and Faraday reactions [27]. In a previous study, the effect of DMSO, as an additive to an alkaline electrolyte, on Zn dissolution and the kinetics of redox reactions during charge/discharge in ZAFBs was investigated. When DMSO was used, passivation of the anode surface reduced due to the suspended ZnO particles that were generated during the discharge process. Moreover, when DMSO was present in the electrolyte, a 41% increase in Zn dissolution and 20% increase in discharge capacity of the ZAFBs were observed [16]. In another study, SDS was added to an alkaline electrolyte and its influence on the performance of ZABs was investigated [18]. Consequently, SDS was able to increase the specific discharge capacity of the ZABs by 24%.

The sulfur content of strong oxidant chemicals like potassium persulfate (KPS, K_2_S_2_O_8_) reacts with alkaline electrolytes and produces sulfate radicals (${\mathrm{SO}}_{4}^{\bar{.}}$), catalyzing the Fenton reaction, as in Equation (1):

(1) $$S_{2}O_{8}^{2-}+H_{2}O\to {\mathrm{HSO}}_{4}^{-}+{\mathrm{SO}}_{4}^{\bar{.}}+{\mathrm{HO}}^{.}$$

When KPS is added to alkaline solutions, it decomposes thermally via a symmetrical rupture of the 0-0 bond, forming a first-order reaction which is catalyzed by hydrogen ions. As a result, sulfate free-radicals are generated due to an active and reversible redox additive which reacts with water, inducing oxygen evolution [28]. KPS, therefore, can accelerate the catalytic oxidation of ${\mathrm{OH}}^{-}$ ions and improve the charge process accordingly [27]. Moreover, some of the ${\mathrm{SO}}_{4}^{\bar{.}}$ ions react with ${\mathrm{OH}}^{-}$ to form ${\mathrm{SO}}_{4}^{2-}$ and then dissolve in the KOH electrolyte. Consequently, a lower amount of ${\mathrm{SO}}_{4}^{2-}$ remains in the solution and is reduced to $S_{2}O_{8}^{2-}$ during the discharge process, indicating a slow discharge rate. It is evident that the use of KPS in alkaline electrolytes can effectively regulate the activity of the reaction in the charge/discharge process. The influence of the KPS additive to the KOH electrolyte was previously investigated on the charge/discharge performance of CoO-supercapacitors; a fast-charge/slow-discharge and long cycling stability was reported [27].

In this study, KPS is proposed as an electrolyte additive in 7 M KOH solution in order to improve the cycle life of rechargeable ZAFBs. Using KPS, the generation and deactivation of free ${\mathrm{SO}}_{4}^{2-}$ radicals by an electrochemical reaction can effectively enhance redox reactions. Moreover, an abundant and strong sulfur-based oxidizing agent can promote the charge transfer of the electrolyte solution and improve the charge/discharge process of ZAFBs. This paper investigates the influence of KPS concentrations on the electrochemical properties of the electrolytes as well as the charge/discharge performance and stability of the ZAFBs. Herein, electrochemical measurements including potentiodynamic polarization (Tafel), cyclic voltammetry (CV) and electrochemical impedance spectroscopy (EIS) have been carried out in order to analyze the electrochemical behavior of the KPS/KOH solution. X-ray diffraction (XRD) has also been employed to study the effect of cycling, in the presence of various electrolytes, on the characteristics of the Zn anode surface. Battery testing was carried out to investigate the performance of the battery under various conditions. This research focuses on providing a promising functional electrolyte, containing a small amount of a sulfur-based oxidizing agent, in order to develop long-life rechargeable ZAFBs having high cycling stability.

## 2. Results and Discussion

### 2.1. Electrochemical Analysis

**Figure 1:**
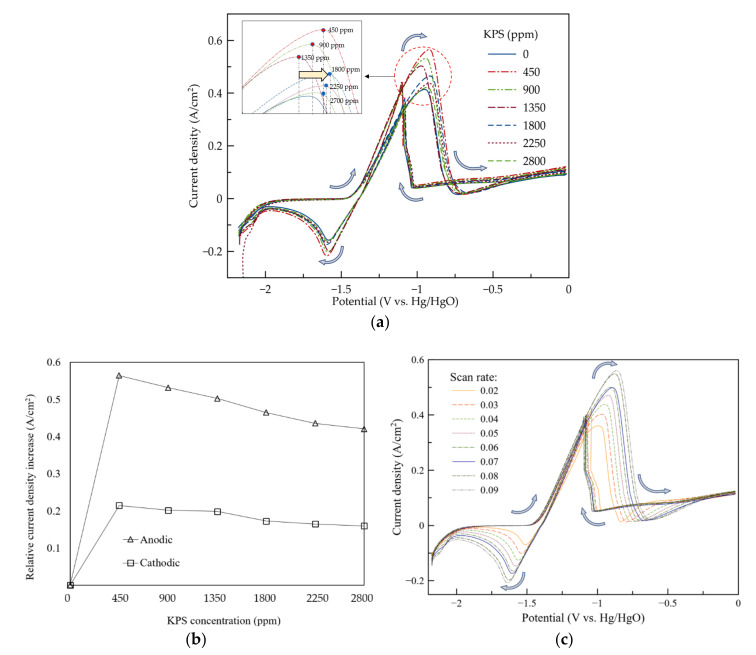
(**a**) Cyclic voltammograms of Zn in different concentrations of KPS at the scan rate 0.07 V/s. (**b**) The increment of current density in anodic and cathodic peaks of the electrolytes in different concentrations of KPS relative to the additive-free electrolyte. (**c**) Cyclic voltammograms of Zn in 450 ppm KPS at various scan rates (0.02, 0.03, 0.04, 0.05, 0.06, 0.07, 0.08 and 0.09 V/s).

In Figure 1a, the results of the CV are shown. It is noted that for all the results, a comparable mechanism of Zn dissolution/deposition occurred. At lower concentrations of KPS (<450 ppm), the results of the CV did not show any significant improvement in the anodic/cathodic reactions (Supplementary Figure S1).

According to Figure 1a, as the concentration of KPS increased, in the positive scan (70 mV s^−1^), an anodic current peak appeared in the voltage range between −1.5 and −1.1 V (vs. Hg/HgO). This broad peak is assigned to the electrochemical oxidation of Zn, generating a zincate complex, according to Equation (2):

(2) $$\mathrm{Zn}+4{\mathrm{OH}}^{-}↔\mathrm{Zn}{\left(\mathrm{OH}\right)}_{4}^{2-}+2e^{-}$$

It is worth noting that at a low concentration of the sulfur-based oxidant (450 ppm KPS), the current density at the anodic peak increased to 0.57 A/cm^2^. The higher current density suggested that the enhanced kinetics of Zn oxidation along with the formation of zincate ions occurred in the forward scan. However, when a higher concentration of KPS (above 900 ppm) was introduced, the anodic current peak was seen to decrease gradually. The presence of KPS, as an oxidant, in a higher concentration, further accelerated the oxidation of Zn, forming Zn^+2^ ions. Therefore, at some point, the concentration of zincate ions in the electrolyte at the vicinity of the anode exceeded its solubility limit. Thus, the conversion of $\mathrm{Zn}{\left(\mathrm{OH}\right)}_{4}^{2-}$ to ZnO duly occurred, as in Equation (3) [29]:

(3) $$\mathrm{Zn}{\left(\mathrm{OH}\right)}_{4}^{2-}↔4{\mathrm{ZnO}}_{\left(s\right)}+H_{2}O+2{\mathrm{OH}}^{-}$$

The produced ZnO then precipitated on the Zn electrode surface and formed a passive layer, followed by the depletion of ${\mathrm{OH}}^{-}$ on the electrode/electrolyte interface. As soon as the thick layer formed on the surface of the anode, the current density decreased sharply. At higher concentrations of KPS (above 1800 ppm), a slight shift to positive potentials was observed in the oxidation peak during the forward scan. The positive shift denotes that the passivation film is more stable and less soluble in the presence of high KPS concentrations. Therefore, a higher over-potential of Zn oxidation is required due to the less active Zn surface directly exposed to the electrolyte [16].

During the negative scan, sharp oxidation peaks were observed around the same potential where the anodic peaks appeared in the forward scan. As reported previously [13], the peaks are assigned to the re-establishment of the Zn oxidation process after breakage of the ZnO passive film and reduction of accumulated ZnO on the surface of the electrode.

In the reverse scan, a cathodic peak, related to the reduction of zincate ions to Zn metal, was observed, as depicted in Equation (4):

(4) $$\mathrm{Zn}{\left(\mathrm{OH}\right)}_{4}^{2-}+2e^{-}\to {\mathrm{Zn}}_{\left(s\right)}+4{\mathrm{OH}}^{-}$$

It is seen that the increase in the anodic current density is comparable to the increase in the cathodic current density (Figure 1b), indicating that 450 ppm KPS promotes the electrodeposition reaction. It is significant that the electrolyte additive at any concentration did not delay or hinder the contribution of HER, around the negative potentials.

In Figure 1c, the effect of the scan rate from 0.02 to 0.09 V/s on the voltammograms of the electrolyte containing 450 ppm KPS is shown. When the scan rate increased, it resulted in rapid electron transfer and thereby fast kinetics of the reaction, which is diffusion-controlled [17]. The potential separation between cathodic and anodic peaks demonstrates the irreversibility of the chemical reaction [30]. In the case of an irreversible reaction, at low scan rates (slower electron transfer), more zincate ions tend to be produced and dissolve in the electrolyte due to the high contact time with hydroxide ions. Consequently, some conversion of $\mathrm{Zn}{\left(\mathrm{OH}\right)}_{4}^{2-}$ to ZnO occurred. The presence of the thick passive layer of ZnO results in a limited oxidation current and therefore smaller anodic peaks.

Supplementary Figure S2 displays the CV results for different concentrations of KPS in the electrolyte in order to determine the electrochemical window, at a scan rate of 0.07 mV/s. The tests were carried out in a three-electrode glass cell. Two platinum (Pt) plates (1 × 1) cm^2^ were used as both the working and counter electrodes. Results revealed that KPS does not have a significant effect on the electrochemical window. Although CV results indicated that some KPS concentrations could increase the reduction currents at the cathodic peaks, they could not improve the electrical window. Thus, the contribution of KPS with regard to the cathodic reactions is not totally clear and requires further analysis.

Potentiodynamic polarization was employed to evaluate the influence of KPS on the corrosion of the Zn anode (pure Zn plate). Corrosion parameters were determined through Tafel analysis. In Equation (5), the anodic and cathodic reactions are described for the small values of the overpotentials [31]:

(5) $$\frac{I}{I_{\mathrm{corr}}}=2.3\left(\frac{\alpha_{a}+\left|\alpha_{c}\right|}{\alpha_{a}\left|\alpha_{c}\right|}\right)\left(E-E_{\mathrm{corr}}\right)$$

where *I*_corr_ is the corrosion current density, α_a_ and α_c_ are the Tafel slopes for anodic and cathodic reactions, respectively, and *E*_corr_ is the corrosion potential. At potentials close to *E*_corr_, Tafel curves can be considered as straight lines. Moreover, the transition resistance between the electrodes and the electrolyte, defined as polarization resistance (*R*_p_), can be determined from the slope of the Tafel curves, as shown in Equation (6) [32]:

(6) $$R_{p}=\frac{\alpha_{a}\left|\alpha_{c}\right|}{2.3I_{corr}\left(\alpha_{a}+\left|\alpha_{c}\right|\right)}$$

In Equation (7), the corrosion rate (CR) for linear polarization can be determined:

(7) $$\mathrm{CR}=\frac{0.13w_{\mathrm{eq}.}I_{\mathrm{corr}}}{d}$$

where $w_{\mathrm{eq}.}$ and d are the equivalent weight and the density of the sample electrode, respectively.

**Figure 2:**
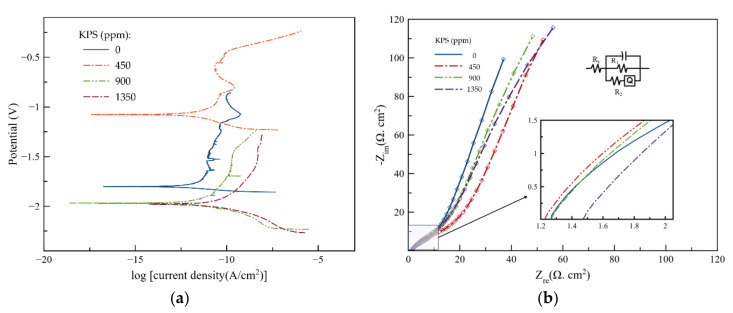
(**a**) Potentiodynamic polarization characteristics of the Zn anode in the electrolytes containing different concentrations of KPS using a scan rate of 0.07 mV/s within the potential range of −0.5 to 0.5 V vs. the open-circuit potential (OCV). (**b**) Nyquist plots of EIS with fitted lines performed at the potential of 0 V in the frequency range from 0.01 Hz to 100 kHz with an AC amplitude of 10 mV of the electrolytes containing different concentrations of KPS.

Potentiodynamic polarization experiments were carried out at 1.66 mV/s within the potential range of −0.5 to +0.5 V vs. the open-circuit potential (OCV). In Figure 2a, the results of potentiodynamic polarization are presented. In Table 1, the corrosion potential (*E*_corr_) and corrosion current density (*I*_corr_) as well as other parameters are listed. When 450 ppm KPS was added to the electrolyte, it was observed that the corrosion potential shifted in a positive direction (from −1.80 to −1.12 V vs. the OCV), while the corrosion current density slightly decreased. Hence, by using 450 ppm KPS, the rate of anodic reactions, viz. HER and Zn dissolution, may be reduced; the corrosion susceptibility (corrosion rate) of the Zn anode will decrease accordingly. A previous study noted similar changes when ethanol was added to an electrolyte solution [17]. When the concentration of KPS increased to 900 and 1350 ppm, corrosion potentials shifted negatively to −1.93 and −2.00 V, respectively. Such an adverse outcome can occur when additional KPS, as a strong oxidant, accelerates Zn dissolution in the electrolyte solution. However, lower *E*_corr_ implies that hydrogen evolution occurs at more negative potentials, improving charge efficiency when used in ZABs. It is observed that the addition of KPS to the electrolyte solution could not significantly lower the polarization resistance. Yet, in the presence of the sulfur-based additive, the corrosion rate is seen to be effectively reduced. Tafel analysis reveals that 450 ppm KPS has a positive effect on the corrosion behavior of the Zn anode and the inhibition of Zn dissolution in the electrode, as it decreases *E*_corr_ and *I*_corr_ as well as the corrosion rate.

The EIS technique was carried out for the various electrolytes containing KPS additives (0–1350 ppm) in order to determine the solution resistance (R_s_) and charge transfer resistances (R_1_ and R_2_). The constant-phase element (Q) denotes the capacitance of the electrode/solution interface. Measurements were performed at a frequency range from 100 kHz to 0.01 Hz with an alternate current (AC) amplitude of 10 mV around the OCV. Nyquist plots were used to fit the EIS data from the equivalent circuit. Results are shown in Figure 2b and Table 2.

Solution resistance (R_s_) slightly decreased when 450 ppm KPS electrolyte was used. This indicates that the low concentration of the sulfur-contained oxidant enhanced the ionic concentration of the aqueous solution at the electrode/electrolyte interface via accelerating the kinetics of Zn dissolution. However, a higher concentration of KPS (900 and 1350 ppm) increased R_s_ as it induced the formation of the passive layer of ZnO over the Zn surface. This proved to be in agreement with the results of the CV, as discussed previously (Figure 1a). In the presence of 450 ppm KPS, the charge transfer resistances (R_1_ and R_2_) significantly increased, implying that KPS in the electrolyte can improve corrosion resistance, decrease the corrosion rate and increase electrolyte conductivity. This outcome is in good agreement with the Tafel polarization curves obtained in the present study (Figure 2a).

It is noted that the electrolyte containing a higher concentration of KPS (>450 ppm) exhibited a sudden drop in the values of R_1_. The presence of a strong oxidant in higher concentrations can result in a higher rate of anodic reactions and Zn dissolution as well as the formation and precipitation of ZnO, as an insulating layer on the surface of the anode. In essence, both lower charge transfer resistance and faster surface reactions are considered an advantage in the charge/discharge processes.

### 2.2. X-ray Diffraction (XRD) Analysis

**Figure 3:**
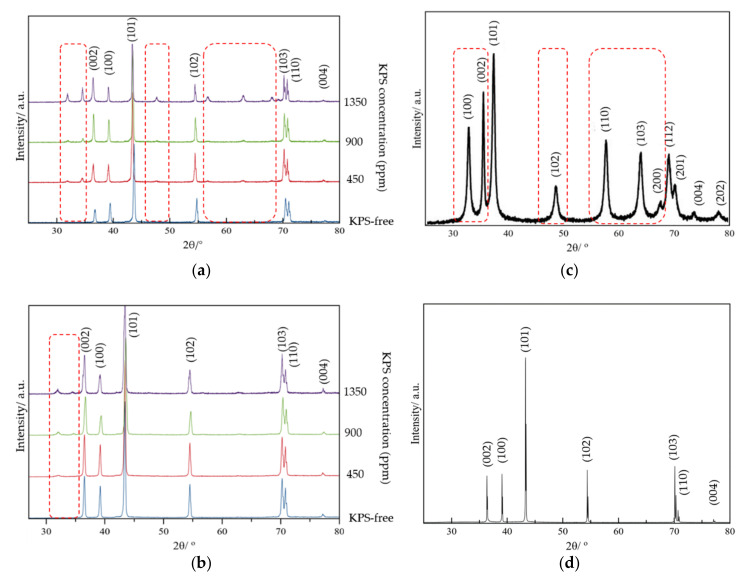
XRD patterns of (**a**) the Zn surface after 8 h immersion in electrolyte solution and 10 charge/discharge cycles at a current density of 15 mA/cm^2^ at the state of discharge; (**b**) at the state of charge; (**c**) ZnO nanoparticles [33]; and (**d**) fresh pure Zn plate.

X-ray diffraction (XRD) was employed to study the effect of the electrolyte additive on the characteristics of the Zn anode surface after 8 h immersion in the electrolyte solution, followed by the performance of 10 charge/discharge cycles, at a constant current density of 15 mA/cm^2^. In Figure 3a,b, the XRD patterns at the states of charge and discharge are shown. In Figure 3c,d, XRD patterns of ZnO nanoparticles and pure Zn plate are displayed.

For both XRD patterns in Figure 3a,b, the diffraction peaks at approximately 36.3°, 38.0°, 43.2°, 54.0°, 71.2°, 72.0° and 77.6° indicate the crystal planes of Zn: (002), (100), (101), (102), (103), (110) and (004), as shown in Figure 3d. After 10 cycles and at the state of discharge, small peaks are seen to appear at 32.2°, 34.0°, 47.6°, 57.1°, 63.5° and 68.0° for the samples containing KPS, followed by a decline in the intensity of the peaks at 39.0°, 43.2° and 54.0°. The new peaks correspond to the diffraction peaks (100), (002), (102),(110), (103) and (200) of ZnO nanoparticles, as shown in Figure 3c [33]. This reflection is assigned to the deposition of ZnO particles on the Zn surface of the anode. The intensity of peaks is found to be relatively smaller at the state of charge. This can be due either to a proportion of ZnO having weaker bonds to the Zn surface, being reduced into Zn^2+^ ions, or to the ZnO surface being covered by the Zn metals, which have been deposited on the anode surface during the charge.

As for the samples having a higher concentration of KPS, the magnitude of the formed/declined peaks was found to be larger/smaller.

### 2.3. Performance of Rechargeable ZAFBs

**Figure 4:**
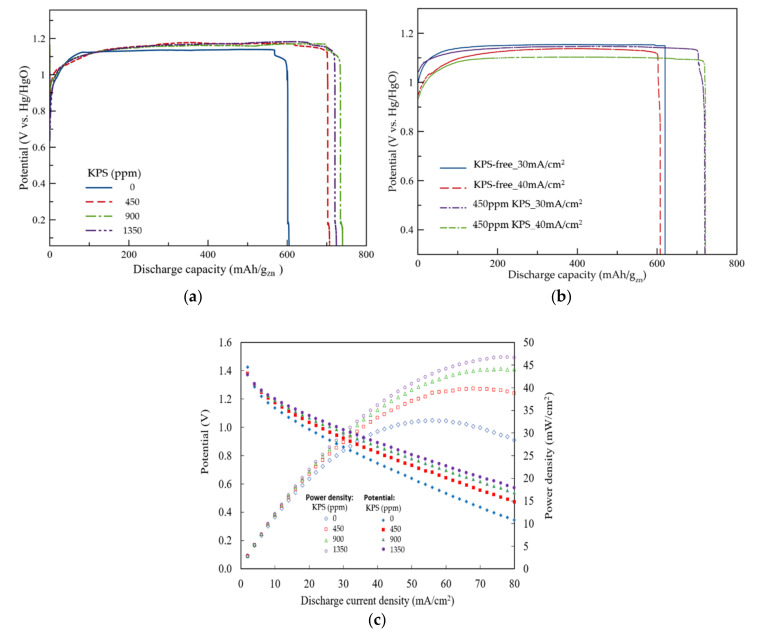
Performance of the zinc–air flow batteries (ZAFBs): (**a**) galvanostatic discharge profiles of the batteries, using different electrolytes, having an electrolyte circulation rate of 100 mL/min at a current density of 15mA/cm^2^ and (**b**) galvanostatic discharge profiles of the batteries containing 0 and 450 ppm KPS at current densities of 30 and 40 mA/cm^2^. (**c**) Polarization characteristics of the batteries, using different electrolytes, having an electrolyte circulation rate of 100 mL/min.

Electrochemical performances of ZAFBs using electrolytes containing 450, 900 and 1350 ppm KPS were evaluated. The electrolyte circulation rate remained constant at 100 mL/min for all experiments. Galvanostatic discharge profiles of ZAFBs, at a constant discharge current density of 15 mA/cm^2^, were obtained using different electrolytes, as shown in Figure 4a.

The discharge profiles, for all cases, were found to be similar to the typical discharge profiles for ZAFBs, as previously reported [34,35]. Although KPS did not significantly affect the discharge potential, it was found to have a substantial impact on improving the discharge time and the capacity of the ZAFBs. The discharge voltage in all cases demonstrated almost similar trends and remained constant at 1.13 V. The batteries containing no additives yielded a specific capacity of 600 mAh/g_zn_. Gradually, specific capacity improved in the presence of higher amounts of KPS in the electrolyte solution. When 450 ppm KPS electrolyte was used, the specific capacity of the batteries improved by 19% (reaching 710 mAh/g_zn_). A further increase in KPS concentration to 900 and 1350 ppm demonstrated an improvement in the specific capacity of 22% and 24%; thus, specific capacity increased to 730 and 745 mAh/g_zn_, respectively. As shown in Figure 4b, when the experiments were repeated with higher current densities of 30 and 40 mA/cm^2^ for the cells containing 0 and 450 ppm KPS, further improvement in capacity was obtained. In the presence of KPS, capacity values improved from 590 to 690 mAh/g_Zn_ (~17%) and from 570 to 690 mAhg_Zn_ (~21%), respectively.

The sulfate ions (${\mathrm{SO}}_{4}^{2-}$), which were generated due to KPS decomposition in water, reacted with ${\mathrm{OH}}^{-}$ and depleted the amount of hydroxide ions in the vicinity of the anode [36]. Hence, the discharge process was seen to slow down and the overpotential increased [26]. However, in the presence of a higher oxidant additive in the electrolyte, lower improvement in capacity was achieved because of the partial formation of ZnO and the consequential passivation of the Zn surface. According to the theoretical specific capacity of Zn, i.e., 819 mAh/g [37,38], the battery containing 450 ppm KPS was seen to use relatively less active material (~13%) than its theoretical value. However, consumption of the active material was found to be 23.3% higher than the additive-free electrolyte at the 15 mA/cm^2^ discharge current density. Such an increase in consumption can be attributed to the partial formation of a passive layer on the Zn anode surface, limiting its utilization during discharge.

In Figure 4c, the cathodic polarization characteristics of the ZAFBs, using different electrolytes, having an electrolyte circulation rate of 100 mL/min are presented. The corresponding power densities are also presented. The similar polarization characteristics of the batteries imply that a similar mechanism for reactions occurs in all cases. Generally, when the discharge current density increases, the discharge voltage decreases linearly due to ohmic losses dominating the cell performance [39]. However, in the presence of KPS, higher discharge potentials were observed at higher current densities, indicating that KPS could improve the polarization characteristics of the batteries. All batteries exhibited an OCV of about 1.40 V. At the current density of 80 mA/cm^2^, the potentials are seen to decrease in the order: 0.33, 0.46, 0.52 and 0.58 V for the electrolytes containing 0, 450, 900 and 1350 ppm KPS, respectively. Consequently, the corresponding power densities: 28, 39, 44 and 47 mW/cm^2^, were obtained.

**Figure 5:**
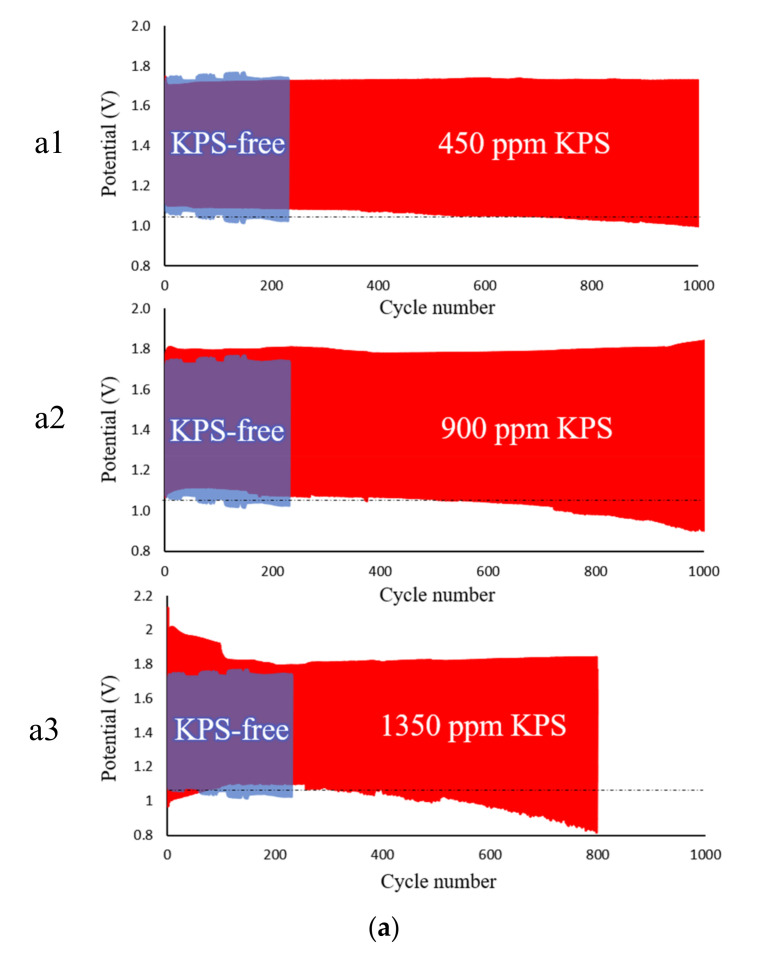

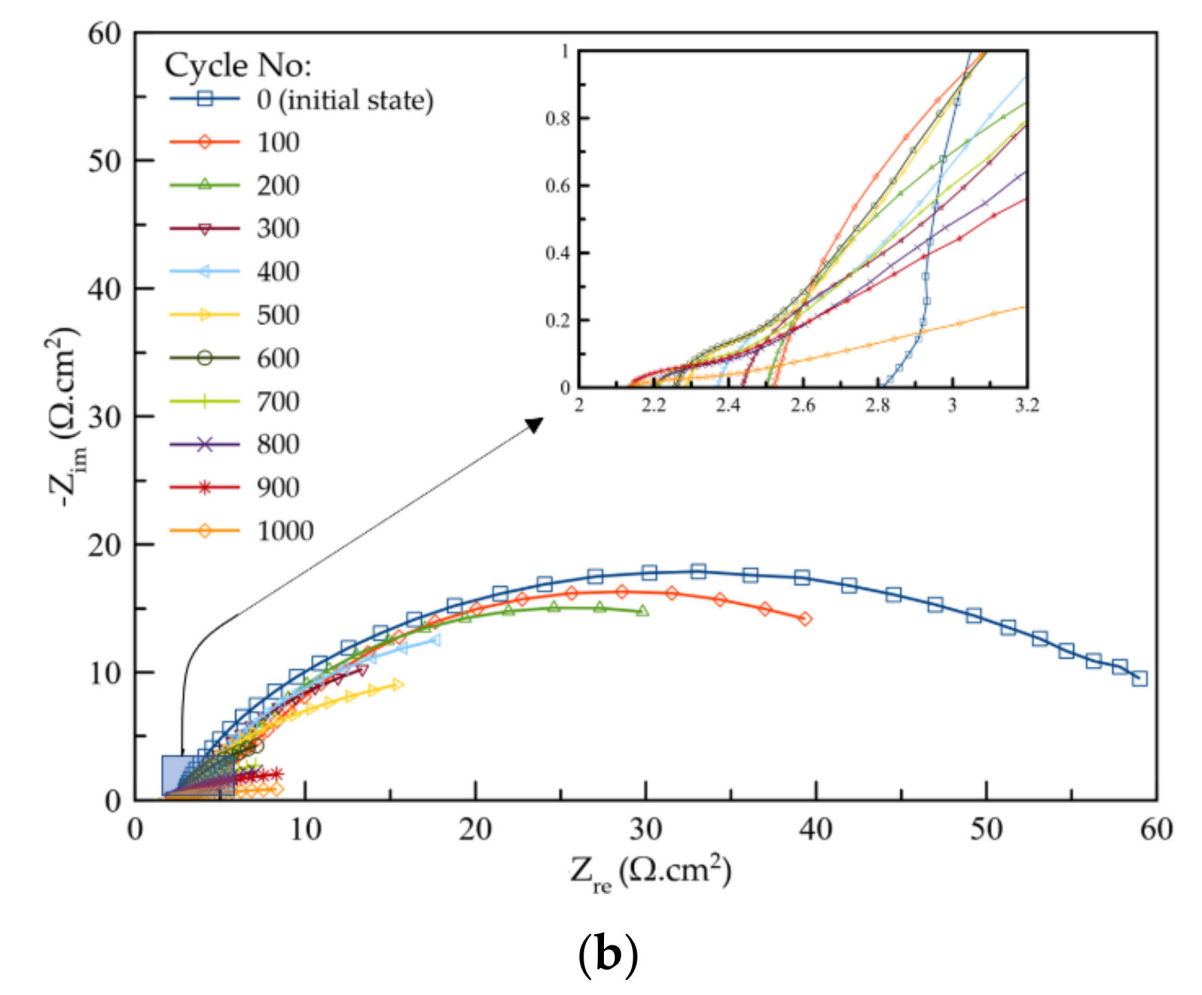
(**a**) Galvanostatic charge/discharge profiles of the batteries, using different electrolytes, having a circulation rate of 100 mL/min and repeated cycling discharge of 50 mA/cm^2^ to 5 mAh and charge of −30 mA/cm^2^ to 5 mAh. (**b**) Nyquist plots of the cycling impact on the impedance of a cell containing 450 ppm KPS additive to the KOH/ZnO electrolyte at initial state and after every 100 charge/discharge cycles until 1000th cycle, in the frequency range from 100 kHz to 0.1 Hz, having an alternate current amplitude of 10 mV around the OCV.

In Figure 5a, the results of the charge/discharge performance of the ZAFBs having different electrolytes are shown. For all experiments, an electrolyte circulation rate of 100 mL/min was maintained. To facilitate initial charging, 0.5 M ZnO was added to each electrolyte. The batteries were set to discharge at 50 mA/cm^2^ to 5 mAh and charge at –30mA/cm^2^ to 5 mAh. In each section of Figure 5a (a1–a3), the results of the charge/discharge performance are demonstrated and are overlapped with the result of the KPS-free electrolyte, for better presentation.

The rechargeability of the batteries was evaluated cyclically until a sharp decline in potentials was observed, denoting that the batteries had been fully degraded. In all cases, except for 1350 ppm KPS, the initial discharge potential was seen to be relatively close to 1.13 V which is similar to the potential, as previously observed (Figure 4a). Furthermore, as the number of cycles increased, discharge potentials declined. After 130 cycles, the battery without KPS started to degrade and fully died after 220 cycles. However, as observed in Figure 5a (a1), the battery containing 450 ppm KPS exhibited a highly stable charge/discharge voltage for 800 cycles. Thereafter, the discharge potential of the battery started to decline, and the battery was fully degraded after 1000 cycles. When 900 ppm KPS was used (Figure 5a (a2)), some instability in the charge/discharge potentials followed for the first 200 cycles, after which cycling remained steady until the 600th cycle. At the initial 100 cycles, the battery containing 1350 ppm KPS performed poorly in terms of stability and exhibited high charge and low discharge potentials (Figure 5a (a3)).

The instability of rechargeable ZABs at initial cycles, in the presence of the high concentration of KPS, is attributed to the formation of a passivation layer which reduces the active surface of the electrode and further depletes ion conductivity. However, when the forced circulation of electrolytes occurred in the flow batteries, the corresponding momentum induced by the flow can reduce the effect of concentration polarization, which eventually limits the passivation. The battery, therefore, after a short period, achieves stable cycling. The charge potentials for 900 and 1350 ppm KPS were about 1.8 and 2 V, respectively which proved to be higher than 1.75 and 1.70 V corresponding to the KPS-free electrolyte and the electrolyte containing 450 ppm KPS, respectively. Thus, the high charge voltage of the batteries containing a high concentration of electrolyte additives is seen to be in agreement with the currents of the cathodic peaks in CV (Figure 1a). The results of the charge/discharge performance indicated that the low concentration of KPS, as an electrolyte additive, can improve the stability of rechargeable ZAFBs. Besides, the batteries containing 450 ppm KPS demonstrated a highly stable charge/discharge voltage that could perform a high number of cycles, without significant loss of performance.

A similar battery configuration was used to perform cycling in the presence of 450 ppm KPS additive where the battery was set to charge at −30 mA/cm^2^ to 5 mAh and discharge at constant 50 mA to the cut-off voltage of 0.8 V. For 800 cycles, the average coulombic efficiency was found to be 91 ± 3%.

To evaluate the cycling impact on cell impedance after each 100th cycle and until the 1000th cycle, Nyquist curves were plotted. For the purpose of cycling, multicycle CV was implemented at a scan rate of 0.07 mV/s, at vertex potentials of −0.5 and 0.5 V vs. the OCV. A cell containing the electrolyte KOH/ZnO with the KPS additive was used. Two pure Zn plates were used as working and counter electrodes. An open cell was employed to ensure that the generated hydrogen gas consistently discharged from the cell, thereby not imposing additional resistance at the electrode/electrolyte interface. After every 100 cycles, EIS was performed in the frequency range: 100 kHz to 0.1 Hz, with an alternate current amplitude of 10 mV around the OCV. This was carried out in order to determine the effect of cycling on the internal resistance of the cell. In Figure 5b, the Nyquist plots for the electrolyte containing 450 ppm KPS are presented and in Table 3, and the related resistance values are listed. In Supplementary Figure S3a,b, the Nyquist plots for the cells containing 900 and 1350 ppm KPS are presented and the related resistance values are listed in Supplementary Tables S1 and S2.

It is noted that the value of R_s_ remained low (<3Ω), suggesting high cycling stability. R_s_ denotes both the resistance of the electrolyte solution and the penetration of the solution within the cathode, anode and separator [40]. During cycling, R_s_ decreased gradually, indicating that the electrolyte penetration was enhanced. Likewise, with cycling, the R_ct_ decreased gradually for 600 cycles. However, after the 600th cycle, R_ct_ began to increase, denoting the fact that the cathode was losing its performance and the battery was consequently degrading. The lower R_ct_ depicts the improvement in ion transfer and reversibility through charge/discharge evolution. As the number of cycles increased, the decrease in R_ct_ can be ascribed to the breakage of the passivation layer and an increment in the active surface area of the electrode.

## 3. Materials and Methods

### 3.1. Electrode and Battery Fabrication

**Figure 6:**
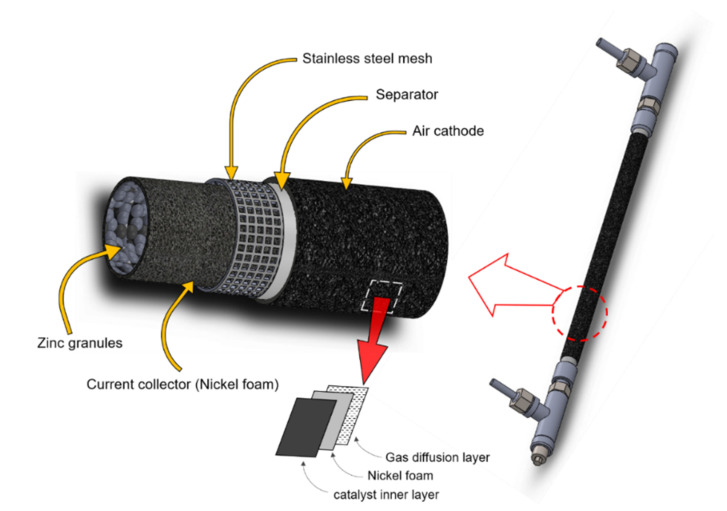
Schematic view of the Zn–air flow battery and its components.

**Figure 7:**
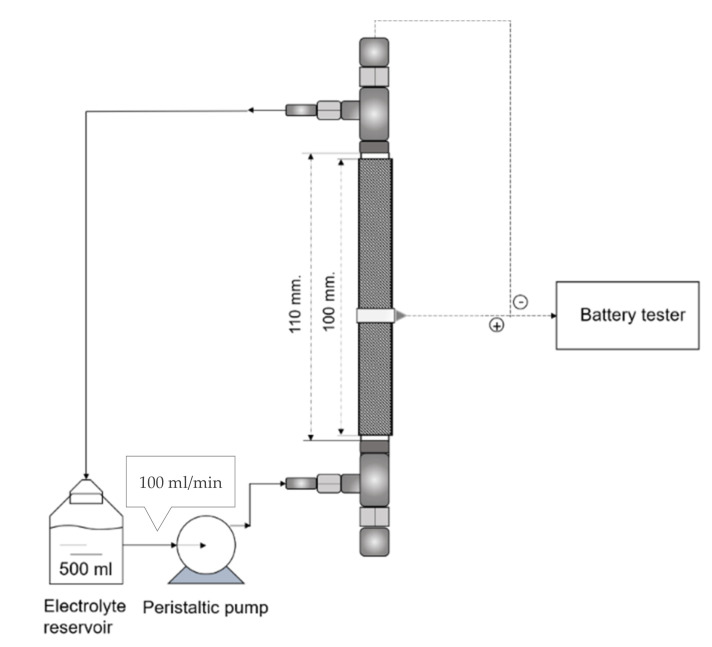
Flow diagram of the Zn–air flow battery.

In the fabrication of ZAFBs, a tubular-structured battery frame made from stainless steel was used. In Figure 6 and Figure 7, a schematic flow diagram and the dimension of the battery are shown. As for anode materials, Zn granules (99.9% pure, average diameter 0.8 mm, Sirikul Engineering Ltd., Samut Prakan, Thailand) were used. A Ni foam sheet (99.97% pure, 1 mm thick and 100 PPI, Qijing Trading Co., Ltd., Fuzhou, China), having an area of (5 × 12) cm^2^, was used as the current collector inside which the Zn granules were packed. The anode was then placed inside the battery frame.

To prepare the separator, 2 g of polyvinyl acetate (PVAc), (TOA Paint Public Co., Ltd., Samut Prakan, Thailand) was coated on both sides of a filter paper (Whatman No.1, 125 mm, Sigma-Aldrich, St. Louis, MO, USA), applied twice on each side, and dried in an oven at 60 °C for 7 min. The separator was cut having an area of (5 × 12) cm^2^ and its thickness was adjusted to 0.2 mm. Then, the separator was wrapped around the stainless-steel mesh section of the battery frame.

In the preparation of the air cathode, the Ni foam sheet, 5 × 10 cm^2^, was used as the current collector. The inner side of the Ni foam sheet was coated with a bifunctional catalyst and its outer side was coated with a gas diffusion layer (GDL). As for the GDL, a mixture of hydrophobic Teflon material (PTFE, 1 μm, Sigma-Aldrich) and carbon blacks (AB-50, IRPC Public Co., Ltd., Bangkok, Thailand) having a ratio of 70:30 in 10 mL ethanol was prepared. The well-stirred solution was coated on one side of the Ni foam sheet and then left to dry. Next, it was hot-pressed at 350 °C for 15 min. To prepare the bifunctional catalyst, alfa-manganese (IV) oxide (α-MnO_2_) (99.99% pure, 5 μm, Sigma-Aldrich) was mixed with carbon blacks (BP2000, Cabot Corporation) at the ratio of 70:30 wt.%. After that, a solution of polystyrene-co-butadiene (5 wt.% of the dry solid) as a binder in 9 mL/g_dry-solid_ toluene (both purchased from Sigma-Aldrich) was added to the catalyst mixture. The solution was well-stirred and applied two times onto the other side of the Ni foam sheet, which was left to dry and hot-pressed at 150 °C for 15 min. Finally, the thickness of the air cathode was adjusted to 0.5 mm using a hot rolling press machine and the cathode was wrapped around the separator. An electrolyte solution was also prepared by dissolving KOH pellets (99% pure, CT Chemical Co., Ltd., Bangkok, Thailand) in de-ionized water (DI water) to obtain the concentration of 7 M. Next, KPS (99% pure) was added to the electrolyte in different concentrations (limited to the solubility of KPS in water, i.e., 4.49 g/100 mL at 20 °C).

### 3.2. Electrolyte Preparation

For the purpose of electrochemical characterization, an electrolyte solution was also prepared by dissolving KOH pellets (99% pure, CT Chemical Co., Ltd.) in de-ionized water (DI water) to obtain the concentration of 7 M. Next, an additive solution consisting of KPS (99% pure) in DI water, having 4.49 g/100 mL concentration, which corresponds to the maximum solubility of KPS in water at 20 °C, was prepared. For the total volume of 20 mL, the concentration of KPS in the electrolyte (ppm) was regulated, according to the volume of KPS solution to be added to the electrolyte solution. Electrolytes containing different concentrations of KPS: 450, 900, 1350, 1800, 2250 and 2800 ppm, were prepared using 1, 2, 3, 4, 5 and 6 vol.% of the KPS solution, respectively. For battery testing, similar electrolytes to those described above were prepared in a total volume of 500 mL with an addition of 0.5 M ZnO.

### 3.3. Characterization and Measurement

Electrochemical techniques including CV, EIS and Tafel were carried out using a potentiostat unit (PAR VersaSTAT 3A, Ametek Inc., Berwyn, PA, USA). An electrochemical cell, 25 mL in volume, was used having 3 electrodes for the purpose of electrochemical characterization. As for all the characterization techniques, pure Zn plate and Pt were used as the working and counter electrodes, respectively. An electrode of mercury/mercury-oxide (Hg/HgO) was also used as a reference electrode.

For the purpose of battery testing, as well as the measurement of discharge capacity and voltage–current polarization, battery testing equipment (NEWARE, CT-4008-5V20mA, Neware Technology Ltd., Shenzhen, China) was used. The batteries were all tested at ambient condition and the circulating electrolyte was maintained at the rate of 100 mL/min throughout all the experiments.

## 4. Conclusions

This paper investigated the effect of KPS as an electrolyte additive on the charge/discharge performance of rechargeable ZABs. In CV analysis, KPS at a low concentration (450 ppm) was found to improve the kinetics of the oxidation reaction by accelerating Zn oxidation and zincate formation. However, at a high concentration of KPS, excess zincate ions were generated in the electrolyte at the vicinity of the anode. As a result, zincate ions converted into ZnO which precipitates on the electrode, reducing the active surface accordingly. The results of potentiodynamic polarization revealed that 450 ppm KPS reduced the corrosion of Zn by 56%. As revealed in the EIS analysis, KPS enhanced the ionic concentration of the aqueous solution at the electrode/electrolyte interface through accelerating the kinetics of Zn dissolution. Results of both Tafel and EIS analyses demonstrated that 450 ppm KPS in the electrolyte improved corrosion resistance and increased electrolyte conductivity. When KPS was used in the rechargeable ZAFBs, it was seen to enhance discharge time as well as the capacity of the batteries. In addition, the battery containing 450 ppm KPS exhibited the highly stable charge/discharge voltage for 800 cycles. EIS analysis revealed that KPS promoted not only the cycling stability, but also the ion transfer and reversibility through the charge/discharge evolution. In conclusion, the low concentration of KPS, as an electrolyte additive, can substantially improve the cycle life of rechargeable ZABs, without significant loss of performance.

## Figures and Tables

**Table 1:** Corrosion parameters obtained from the Tafel analysis for the Zn anode in the electrolytes containing different concentrations of KPS.

KPS (ppm) in Electrolyte Solution	*E*_corr_ (V)	log *I*_corr_ (A/cm^2^)	α_a_	$\left|\alpha_{c}\right|$	*R*_p_ (Ω)	CR
0	−1.80	−11.46	0.60	0.68	261.27	6.31
450	−1.12	−11.82	0.28	0.48	518.39	2.76
900	−1.93	−11.16	0.73	0.87	250.74	12.60
1350	−2.00	−10.57	0.64	0.84	59.08	49.01

**Table 2:** Resistance values of Zn anodes in the solution electrolyte containing KPS (450, 900 and 1350 ppm) in 7 M KOH.

KPS Concentration	R_s_ (Ω)	R_1_ (Ω)	R_2_ (Ω)	Q, (S/s^n^)	
				Y_0_ (s^n^/Ω)	0 < n < 1
0	1.26 ± 0.61%	1230 ± 5.95%	4.91 ± 0.77%	5.91 × 10^−4^ ± 1.75%	0.76 ± 0.40%
450	1.22 ± 0.37%	1333 ± 4.83%	7.46 ± 0.44%	4.72 × 10^−4^ ± 1.15%	0.76 ± 0.21%
900	1.27 ± 0.75%	950.3 ± 5.05%	8.02 ± 0.81%	3.72 × 10^−4^ ± 0.98%	0.79 ± 0.32%
1350	1.48 ± 0.54%	856 ± 3.12%	13.69 ± 0.74%	3.29 × 10^−4^ ± 1.98%	0.78 ± 0.40%

**Table 3:** Resistance values of cell impedance after every 100 cycles of CV for the open cell with the electrolyte containing 450 ppm KPS.

Concentration KPS (ppm)		Cycle Number										
		0	100	200	300	400	500	600	700	800	900	1000
**450**	R_s_(Ω) (±1%)	2.81	2.52	2.51	2.45	2.39	2.30	2.28	2.17	2.14	2.12	2.11
	R_ct_ (Ω) (±1%)	8.21	8.05	7.85	7.36	7.02	6.43	6.12	6.37	7.62	8.56	9.44

## Supplementary Materials

The following are available online at https://www.mdpi.com/1422-0067/21/19/7303/s1.

## Abbreviations

ZAB	Zinc–air battery
ZAFB	Zinc–air flow battery
EIS	Electrochemical impedance spectroscopy
CV	Cyclic voltammetry
XRD	X-ray diffraction
DI water	De-ionized water
CR	Corrosion rate
OCV	Open-circuit voltage
AC	Alternative current
GDL	Gas diffusion layer
HER	Hydrogen evolution reaction
R_s_	Solution resistance
R_ct_	Charge transfer resistance
