# Supplementary material for: Enhanced Cycling Performance of Rechargeable Zinc–Air Flow Batteries Using Potassium Persulfate as Electrolyte Additive
Source: Int J Mol Sci. 2020 Oct 2;21(19):7303. doi: 10.3390/ijms21197303 (PMC7582734; doi:10.3390/ijms21197303)
Supplement: Supplementary file 1 [file ijms-21-07303-s001.pdf]

Supplementary Materials

Enhanced Cycling Performance of Rechargeable Zinc–Air Flow Batteries Using Potassium Persulfate as Electrolyte Additive

Ramin Khezri [1], Soraya Hosseini [1], Abhishek Lahiri [2], Shiva Rezaei Motlagh [3], Mai Thanh Nguyen [4], Tetsu Yonezawa [4,5] and Soorathep Kheawhom [1,6,*]

[1] Department of Chemical Engineering, Faculty of Engineering, Chulalongkorn University, Bangkok 10330, Thailand; ramin.k@chula.ac.th (R.K.); soraya.h@chula.ac.th (S.H.)
[2] Department of Chemical Engineering, Brunel University London, London UB8 3PH, United Kingdom; abhishek.lahiri@brunel.ac.uk
[3] Department of chemical engineering, faculty of engineering, Universiti Putra Malaysia, Selangor 43300, Malaysia; shiva.rezaei@student.upm.edu.my
[4] Division of Materials Science and Engineering, Faculty of Engineering, Hokkaido University, Hokkaido 060-8628, Japan; mai_nt@eng.hokudai.ac.jp (M.T.N.); tetsu@eng.hokudai.ac.jp (T.Y.)
[5] Institute for the Promotion of Business-Regional Collaboration, Hokkaido University, Sapporo 001-0021, Japan
[6] Research Unit of Advanced Materials for Energy Storage, Chulalongkorn University, Bangkok 10330, Thailand
* Correspondence: soorathep.k@chula.ac.th

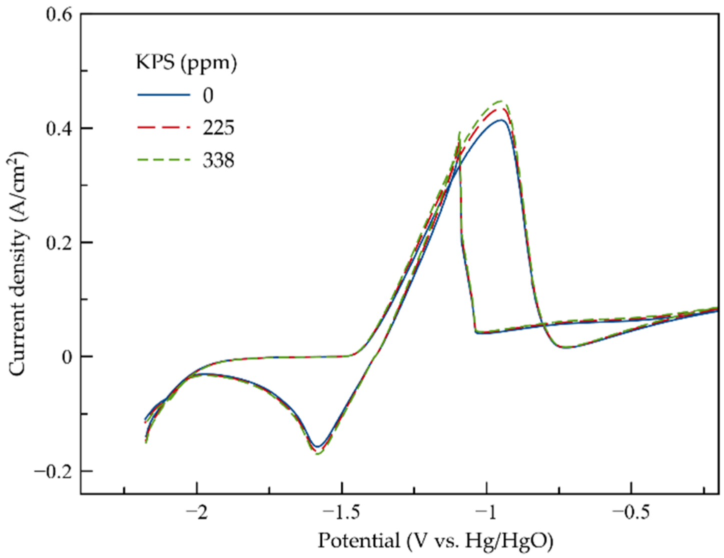

Figure S1. Cyclic voltammograms of Zn in a cell containing 0, 225 and 338 ppm KPS additive at scan rate 0.07 V/s.

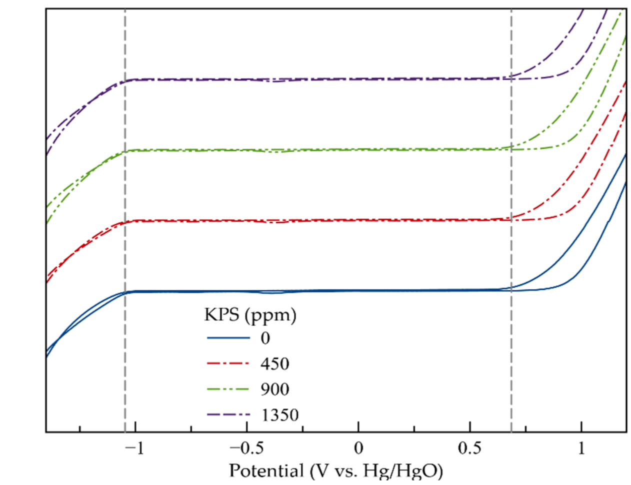

Figure S2. Electrochemical windows in the presence of 0, 450, 900 and 1350 ppm KPS additive.

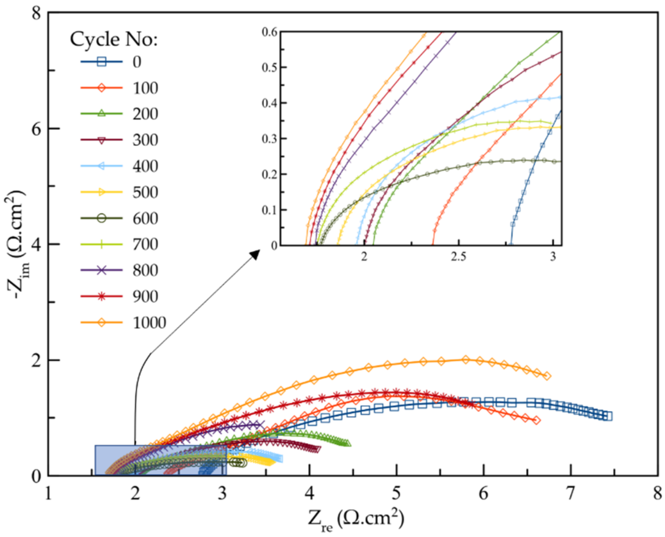

(**a**)

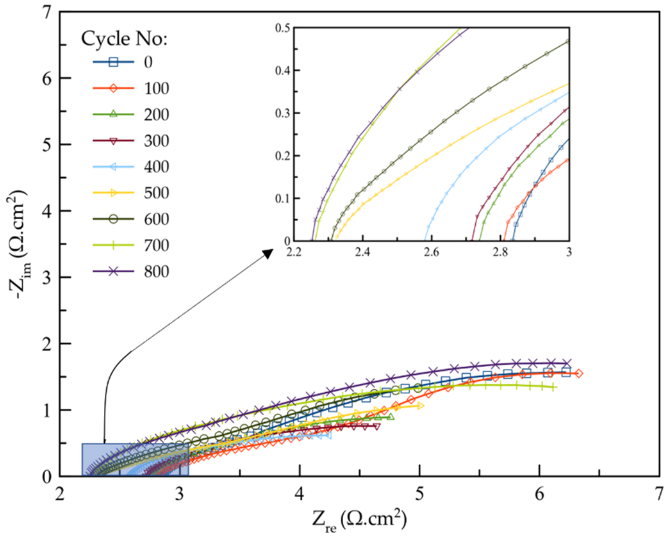

(**b**)

Figure S3. Nyquist plots of the cycling impact on the impedance of a cell containing (**a**) 900 ppm KPS and (**b**) 1350 ppm KPS additive to KOH/ZnO electrolyte at initial state and after every 100 charge/discharge cycle until the 800th cycle, at a frequency range from 100 kHz to 0.1 Hz, having an alternate current amplitude of 10 mV around OCV.

Table S1. Resistance values of cell impedance after every 100 cycles of CV for open cell with the electrolyte containing 900 ppm KPS.

Concentration KPS (ppm)		Cycle Number										
		0	100	200	300	400	500	600	700	800	900	1000
900	R_s(Ω) (±1%)	2.77	2.36	2.04	2.00	1.95	1.85	1.76	1.75	1.74	1.71	1.69
	R_{ct} (Ω) (±1%)	8.76	7.05	7.93	7.36	7.15	6.89	6.57	6.97	10.92	11.52	13.14

Table S2. Resistance values of cell impedance after every 100 cycles of CV for open cell with the electrolyte containing 1350 ppm KPS.

Concentration KPS (ppm)		Cycle Number										
		0	100	200	300	400	500	600	700	800	900	1000
1350	R_s(Ω) (±1%)	2.83	2.81	2.73	2.71	2.57	2.32	2.30	2.26	2.25	-	-
	R_{ct} (Ω) (±1%)	14.31	13.72	7.75	7.84	8.32	8..86	10.17	13.01	14.41	-	-
